# Latent tuberculosis in children and youth with type 1 diabetes mellitus in Dar es Salaam, Tanzania: a cross section survey

**DOI:** 10.1186/s12879-023-08753-4

**Published:** 2023-10-30

**Authors:** Edna S. Majaliwa, Kandi Muze, Evance Godfrey, Kenneth Byashalira, Blandina T Mmbaga, Kaushik Ramaiya, Sayoki G Mfinanga

**Affiliations:** 1 Kilimanjaro Christian Medical University College, Box 2240, Moshi, Kilimanjaro United Republic of Tanzania; 2 Muhimbili National Hospital, Box 65000, Dar es Salaam, United Republic of Tanzania; 3 Kibong’oto Infectious Diseases Hospital, Sanya Juu, Siha, United Republic of Tanzania; 4 Kilimanjaro Clinical Research Institute, Moshi, United Republic of Tanzania; 5 Shree Hindu Mandal Hospital, Dar es Salaam, United Republic of Tanzania; 6 Muhimbili Medical Research Centre, National Institute for Medical Research, Dar es Salaam, United Republic of Tanzania; 7 School of Public Health, Department of Epidemiology and Statistics, Muhimbili University of Health and Allied Sciences, Dar es Salaam, United Republic of Tanzania; 8 Alliance for Africa Research and Innovation, Dar es Salaam, United Republic of Tanzania

**Keywords:** Diabetes, T1DM, Glycaemic, Latent tuberculosis, Children, Youth, QuantiFERON

## Abstract

**Background:**

Data for latent tuberculosis in patients with type 1 Diabetes in Africa is limited. We assessed the prevalence of latent tuberculosis in youth and children with type 1 Diabetes in Dar es Salaam –Tanzania.

**Methods:**

Our cross-sectional study recruited children and youth with T1DM by stage of puberty, glycaemic control, and age at diagnosis from January to December 2021 in Dar es Salaam. Participants were screened for the presence of latent Tuberculosis using the QuantiFERON test. A positive test was considered to have latent TB.

**Results:**

Of the 281 participants, the mean age was 19 (± 6) years, 51.2% were female, and 80.8% had either a primary or secondary level of education at baseline. The prevalence of latent TB was 14.9% and was slightly higher in females (52.4%) than in males. This difference, however, was insignificant (p > 0.05). On the other hand, the proportion of latent TB was significantly higher in uncontrolled HbA1c levels (76.2%) than in those with controlled HbA1c (23.8%) [p = 0.046]. Duration of diabetes and age at diagnosis did not affect the occurrence of latent Tuberculosis [p > 0.05]. Meanwhile, in the regression model, participants with latent TB were more likely to have uncontrolled HbA1c. [p = 0.045]

**Conclusions:**

Despite the methodological limitations, this survey highlights the high prevalence of latent TB among children and youth with diabetes; shouting for better control. These results clearly show the need to screen for Tuberculosis in children and youth with diabetes and start them on prevention as per protocol, especially in tuberculosis-endemic areas like Tanzania.

## Background

Both tuberculosis (TB) and diabetes mellitus (DM) are increasing worldwide, with asymptomatic presentation when they co-exist, leading to interference in glycaemic control, ant-tuberculosis therapy failure, relapse and death [1, 2]. Some studies (Mixed adult and youth) have shown how frequent TB is in patients with DM, and most of the time, they are asymptomatic [3]. The prevalence of TB in diabetes ranges from 1- 29.3% [1, 4–10]. Studies have shown that diabetes interferes with innate immunity affecting chemotaxis phagocytosis and antigen presentation. It also affects T-cell function making progression from latent to active tuberculosis easy [11]. On the other hand, tuberculosis affects glycaemic control by making it poorer in individuals with diabetes, due to immunological changes as a response to tuberculosis, but also due to drug-drug interactions between Anti-TB and anti-diabetics [11, 12]. Studies have also shown that the life time risk of Latent Tuberculosis Infection (LTBI) to progress to active tuberculosis ranges between 5 and 15% with increased risk in those with immunosuppressive conditions like Diabetes [13, 14]. In developing countries, such as Tanzania where tuberculosis is endemic, the 10-year risk of acquiring tuberculosis may be as high as 25% for diabetes mellitus patients [15]. Some studies have shown that once a patient with diabetes mellitus contracts tuberculosis, it is often a multidrug resistance TB which may result in treatment failure or death [11, 16, 17]. A systematic review of 13 observation studies showed there is an increased risk of latent tuberculosis in diabetes mellitus patients compared to the control [13]. The rate of latent tuberculosis in a patient with diabetes is as high as 42% [18]. The risk is higher when glycated hemoglobin (HbA1C) is more than 7% [12]. Other studies done in children, showed the prevalence of tuberculosis (both latent and active) to be between 4.2 and 29.5%, which is similar to adult population [10, 19, 20]. Most children in sub-Saharan African countries have poor glycemic control HbA1C > 7% [21–23] and since diabetes is increasing in incidence and prevalence in children, the likelihood of latent and active TB is high. Studies done in Cape town showed that 29.8% of children with diabetes had latent/active Tuberculosis [10]. Studies have been done mostly in adults showing the increase of tuberculosis in DM, which might be a new infection or reactivation of the previous disease [19, 20]. Some studies have shown that poor glycemic control is frequent in children with TB [12, 24]. This is a significant challenge in which interventions needs to be set, and progression of both diseases followed up. According to the WHO guideline on management of latent TB there was no recommendation on screening of LTB on T1DM patients due to weak evidence [25]. There is no data that report the prevalence of latent tuberculosis in children and youth with diabetes in Tanzania. Therefore, this study aimed to determine the prevalence of latent tuberculosis- diabetes- co-morbidity among children and youth as well as investigating the factors association with diabetes-tuberculosis co-morbidity, including diabetes complications (nephropathy and retinopathy) among children and Youth in Tanzania setting.

## Methods

### Study design and population

The study employed a cross-sectional study design to establish the presence of latent-TB in type 1 DM(T1DM) participants. Study participants were recruited from the 5 pediatric and youth diabetes clinics located in Dar es salaam. In each study site, all children and youth with T1DM were included in our study. Recruitment of participants started from Jan 2021 – December 2021. The population included all children and youth aged 1–25 years inclusive attending pediatric diabetes clinics (These clinics include children and youth up to 26 years of age) located in Dar es Salaam, children whose parents gave consent, children aged 12-<18 years, who gave assent on top of parental consent and youth who gave consent. We excluded children with co-morbidity like sickle cell disease, children under one year (who may not be type 1 diabetes) and those above 25 years.

### Study setting

The study was conducted in five health facilities with specific pediatric diabetes and youth clinic located in Dar es Salaam. These sites were Mwananyamala in Kinondoni Municipal, Temeke in Temeke Municipal, Vijibweni in Kigamboni Municipal, and Muhimbili in Ilala municipal and one private clinic at Hindu mandal Hospital. Dar es Salaam is the largest city in Tanzania located in the Eastern zone of the country with an estimated population of 5 million inhabitants according to the 2022 census. Dar es Salaam contributes most patients of DM and TB in the entire country. It has a high prevalence of DM in the general population of 9% [26], and had 15% of new Tuberculosis cases diagnosed in 2021 [27].

### Data collection

A structured questionnaire was used to collect the required study information, including socio-demographic factors of children and youth, date of birth, number of admissions, amount and frequency of insulin taken, any other medication (e.g., Ant tuberculous), and complications encountered. Physical examination was done including anthropometric measurements, pubertal assessment and eye check using fundopictures. Assessment for puberty was done using tanner staging then grouped into pre-pubertal, pubertal and post pubertal [28].

The first morning urine of the day was collected in an empty clean bottle. Using a multistrip URIT 14 g (Accurex diagnostic, India) determination of presence of proteins, Microalbuminuria, ketones, and sugar in urine was done. Then Albumin creatinine ratio was calculated. A patient was regarded as abnormal if reading was ≥ 30mcg/gm creatinine.

### Anthropometric

One well-trained research assistant performed all anthropometric measurements including weight, height and pubertal assessment. Weight and height machine (Tanita BC-553 digital 150 kg scale and a portable stadiometer- Seca 214). The weighing scale was firmly put on a flat floor, the child/youth removed his/her shoes and heavy clothes like sweaters, remaining with one light cloth. The child/youth stood at the center of the scale with both feet. Weight was then read from the weighing scale and rounded to the nearest 100 g. The child moved from the weighing scale to the height scale (a portable stadiometer- Seca 214). In addition to footwear removal to measure height, any hair ornaments (e.g., bands, hats, turbans) that could interfere with the measurement were removed. The head of the stadiometer was then raised to allow room for participant to stand underneath. The research assistant checked if the child/youth is standing at the center of the base of the plate, heels together, arms to the sides and legs straight, shoulder relaxed and head positioned in the Frankfort horizontal plane, the patient was instructed to keep their eyes focused straight on a point ahead and to stand as upright as they can, then the head piece was lowered to the highest place of the head with enough pressure to compress the hair. The participant was then told to step off the stadiometer when reading the measurement. The height was recorded to the nearest 0.1 cm. BMI was then calculated using the Quetelet equation (weight in kilograms divided by the square of height in meters).

### Blood pressure

The child /youth was seated on a chair with his/her arm rested on the table. Then the screw was turned to remove the air out of the cuff, which depending on the size of the child was placed on the upper arm above the elbow. The “on” button switched on and the reading appeared automatically on the screen of the machine (Dinamap).

### Eye examination

A nurse trained to do eye examination in patients with diabetes took the fundal pictures which were analyzed by an ophthalmologist. At the start of the examination, the child was examined for visual acuity using Snellen’s chart and standing at 6 m. This was followed by dilating the eyes of the children using one drop of tropicamide eye drop in each eye and left for 10 to 30 min for the pupil to dilate, after which the fundus pictures were taken by a nurse trained in fundo picture techniques, using a fundus Camera (Topcorn TRC 50 EX, Japan). The ophthalmologists read the fundus pictures for accuracy and quality control.

### Screening for tuberculosis

**Fig. 1:**
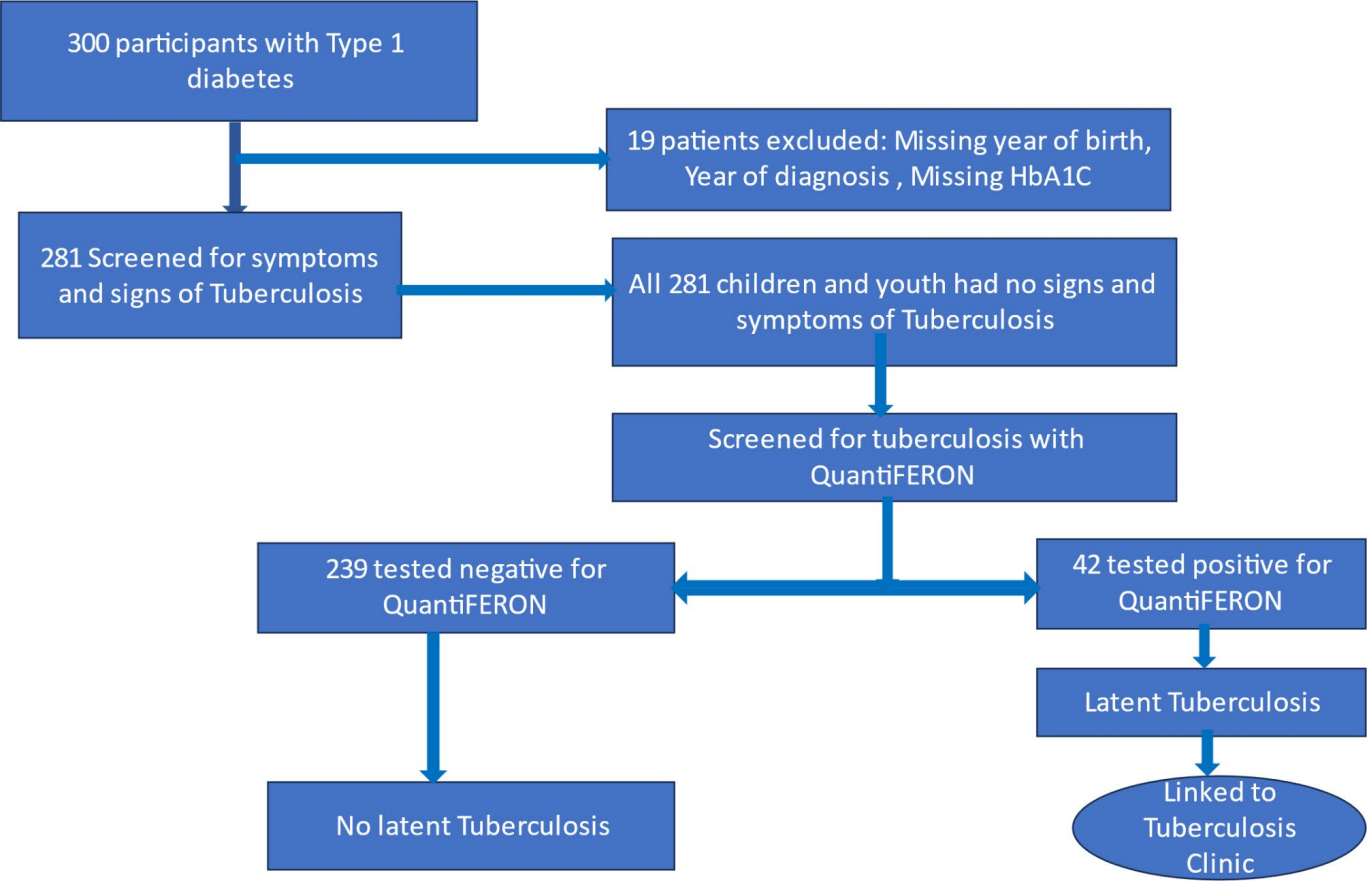
Flow chart in Diabetes clinic: Screening for Tuberculosis

Then children and youth were screened for symptoms of Tuberculosis as per NTLP guideline, if they had TB symptoms, samples for gene- X- pert was taken. All those who did not have symptoms underwent QuantiFERON testing (QuantiFERON TB Gold plus Interferon Gamma Release Assay (IGRA), a whole blood assay developed to detect the IFN-γ produced in vivo by sensitized T cells after in vitro stimulation with mycobacterial antigen and is not affected by environmental mycobacteria or BCG vaccine [29] (Fig. 1). These children and youth were also screened for retinopathy and nephropathy.

### Laboratory investigations

We collected blood samples from the anterior cubital fossa (about 4.5 mls) following standard procedures. We immediately used one drop of blood on the glucostick for determination of random/fasting blood glucose, using a glucometer (Gluconavii, home health, (UK) LTD Unit A, Greatham Road, industrial estate Greatham Road, Bushey, Hertfordshire WD23 2NZ, United Kingdom), another drop was put on Hemocue® HbA1c 501, (full automated point of care machine (Radiometer group, Angelholm-Sweden) for HbA1c determination, after the drop of blood was put on an HbA1C machine, with a wait of 6 min the results displayed on the screen of the machine and was recorded. The remaining 4 mls were put in the QuantiFERON gold bottles, one ml for each bottle (Negative control (Grey), TB1(green), TB2 Yellow and Mitogen (Purple). These samples were taken to the Hindu-mandal hospital on the same day of collection at room temperature in Dar es salaam 25-28^o^C, and incubated at 37^o^C for 24 h. After the 24-hour incubation at the laboratory, the samples were centrifuged to get plasma. Elisa test was performed, absorbance value obtained Using a QuantiFERON analysis software, the results were analyzed. All the samples were taken and analyzed at the Hindu Mandal hospital laboratory except for point of care blood glucose and HbA1c. All the 42 participants with latent tuberculosis were linked to the tuberculosis services in respective hospitals. Quality of care check and standardization of the tests, few samples were taken in both the Shree Hindu Mandal Hospital (The study main laboratory) sample and Muhimbili National Hospital (The main referral hospital in the country) except for the QuantiFERON test which were taken to the main government chemists Laboratory.

### Testing for microalbuminuria and urine creatinine

About 2 mls of the 1st morning spot urine collected in an empty sterilized bottle was used. Using a multistics for microalbuminuria, the strips were URIT 14 g (Accurex diagnostic, India) were dipped into the urine container and weighted for 60 s. Then the color of the uristrips was compared with the color scale on bottle, The strips measured the presence of protein, microalbumin, creatinine, ketone and sugar. Albumin Creatinine ratio was then calculated from the two readings. The minimum sensitivity of the test strip was 30mcg/dl of Albumin in urine.

### Data management and statistical analysis

A structured questionnaire was prepared to ascertain the participants’ demographic and clinical information. Data was analyzed using SPSS version 25 (IBM SPSS, Armonk, NY, USA). Mean with standard deviation (SD) or Median with 25th and 75th interquartile range (IQR) were used to summarize parametric and non-parametric continuous variables (e.g. Age, weight, height, HbA1c levels). Comparison of categorical variables such as frequencies and proportion of children and youth screened and diagnosed with or without latent TB were performed using the Pearson chi-square or Fisher’s exact test. A multivariate logistic regression analysis was used to calculate the odd ratio (OD) with 95% confidence interval (CI) for the risk factors that were associated with latent TB and DM complications such as retinopathy and nephropathy. A p-value of < 0.05 was considered statistically significant.

## Results

### Socio-demographic characteristics of the study participants

A total of 300 children and youth with T1DM were enrolled in the study, of these, 281 (93.7%) were included in the analysis with the exclusion of 19 (6.3%) due to missing data as per (Fig. 1). Of the 281 participants included in the study, 144 (51.2%) were females, and the mean age was 19 (± 6) years (Table 1). A higher proportion of males were significant at post-puberty age compared to females who were significantly either puberty or pre-puberty age at baseline (p = 0.003). Weight of post-puberty participants was significantly higher (51.0 ± 16.0 kg) compared to participants who were either pre or puberty age (27 ± 10 kg) and (42 ± 13 kg)) respectively, p < 0.001. Proportion of uncontrolled HbA1c was higher at puberty age (72%), followed by 67.6% in pre-puberty age, and 58.5% in post-puberty participants. These differences, however, were insignificant (> 0.05). Overall Prevalence of Latent tuberculosis was 14.9%. The Prevalence was 15% in post-puberty participants, 19.6% in pubertal patients and 8.1% in pre pubertal patients. Again, the difference in prevalence across the groups was insignificant (> 0.005) [ Table 1]. TB symptom screening was negative to all participants.

**Table 1:** Socio-demographic characteristics of the study participants by their age groups (N = 281)

Participants Characteristics	Total (%)				
		Pre –pubertal	Pubertal	Post-pubertal	^§^p-value
Age in years ;Median (IQR)	19 (15–24)	8 (6–9)	14 (12– 15)	22 (19– 25)	
Sex					
Male	137 (48.8)	11(29.7)	19 (37.3)	107 (55.4)	0.003
Female	144 (51.2)	26 (70.3)	32 (62.7)	86 (44.6)	
**Anthropometry**					
HT (mean ± SD) cm	153 (± 18)	131 (± 17)	147 (± 14)	159 (± 10)	< 0.001
WT (mean ± SD) Kg	51 (± 16)	27 (± 10)	42 (± 13)	51 (± 16)	< 0.001
HbA1c control*					
< 10% (controlled)	106 (37.7)	12 (32.4)	14 (27.5)	80(41.5)	0.144
≥ 10% (Uncontrolled)	175 (62.3)	25 (67.6)	37 (72.5)	113 (58.5)	
**Duration of Diabetes**					
< 5years	141 (50.2)	21 (56.8)	27 (52.9)	93 (48.2)	0.576
5 Years or above	140 (49.8)	16 (43.2)	24 (47.1)	100 (51.8)	
**Smoking status**					
No	280 (99.6)	37 (100)	51 (100)	192 (99.5)	0.687**
Yes (current and past)	1 (0.4)	0 (0)	0 (0)	1 (0.5)	
**Level of education**					
No formal education	18 (6.4)	7 (18.9)	2 (3.9)	9 (4.7)	< 0.001**
Primary or Secondary	227 (80.8)	30 (81.1)	49 (96.1)	148 (76.7)	
University/college	36 (12.8)	0 (0)	0 (0)	36 (18.7)	
**QuantiFERON test status**					0.342**
positive	42 (14.9)	3 (8.1)	10 (19.6)	29 (15.0)	
Negative	239 (85.1)	34 (91.9)	41 (80.4)	164 (85.0)	

*2 participants not recorded HbA1c results, ** Fisher’s exact test was used

### Comparison of T1DM participants screened for latent TB

The table below compares characteristics of 281 participants with or without TB. Overall, 42 (14.9%) of 281 participants screened for latent-TB had tuberculosis. The mean age and weight of participants with latent TB respectively were slightly higher (19 ± 14.0) years, and (52.3 ± 15.6 kg), compared to mean age and weight of participants without latent tuberculosis (19 ± 14.0), and (50.0 ± 15.6 kg). Nonetheless, this difference was insignificant (P > 0.05). A high proportion of participants with latent tuberculosis had no formal education (9.5%) when compared to those without latent TB (5.9%). Even so, this difference was insignificant (> 0.05). Meanwhile, proportion of uncontrolled HbA1c was significantly higher in participants with latent TB (76.2%) versus (60.0%) without latent TB (P = 0.046). On the other hand, duration of DM for more or equal to 5 years was similar among participants with and those without latent TB (p0.05). (Table 2).

**Table 2:** Comparison between children found with and without latent Tuberculosis (N = 281)

Variable		Total N = 281(%)			p-value
			With Latent TB N = 42 (%)	Without Latent TB N = 239 (%)	
Age (mean ± SD) Years		19(± 6)	19 (± 6)	18 (± 6)	0.697
Sex	Male	137 (48.8)	20 (47.6)	117 (49.0)	0.873
	Female	144 (51.2)	22 (52.4)	122 (51.0)	
Anthropometry	Height (± SD) cm	153 (± 18)	154.1 (± 17.3)	153.5 (± 15.6)	0.810
	Weight (± SD) Kg	51 (± 16)	52.3 (15.6)	50.2 (15.8)	0.429
Level of education	No formal education	18 (6.4)	4 (9.5)	14 (5.9)	0.351**
	Primary/Secondary	227 (80.8)	35 (83.3)	192 (80.3)	
	University/college	36 (12.8)	3 (7.1)	33 (13.8)	
HbA1c	< 10% (controlled)	106 (37.6)	10 (23.8)	96 (40.0)	0.046
	≥ 10% (Uncontrolled)	175(62.4)	32 (76.2)	143(60.0)	
Duration of Diabetes	< 5 years	141 (50.2)	23 (54.8)	118 (49.2)	0.616
	≥ 5 Years	140 (49.8)	19 (45.2)	122 (50.8)	
Smoking status	Yes	1 (0.4)	0 (0)	1 (0.4)	0.851**
	No	281 (99.6)	42 (100)	239 (99.6)	

**Fisher’s exact test was used

### Risk factors associated with latent tuberculosis among children and youths with diabetes mellitus

In a multivariate regression model, participants with uncontrolled HbA1c level (OR 2.19, 95% CI 1.02–4.71, p = 0.045) were significantly more likely to be diagnosed with latent TB compared to those participants with controlled HbA1c [Table 3].

**Table 3:** Factors associated with Latent Tuberculosis among children and youths with diabetes mellitus

Variables	Unadjusted OR (95% CI)	*P-value*	Adjusted** OR (95% CI)	*P-value*
**Sex** (Female)	Reference			
Male	0.95 (0.49–1.83)	0.873	0.88 (0.45–1.75)	0.730
**At Pre-Puberty age** (No)	Reference			
Yes	0.47(0.14–1.59)	0.223		
**Puberty Age** (No)	Reference			
Yes	1.52 (0.69–3.33)	0.299	2.75 (0.69–10.91)	0.768
**Post-Puberty Age** (No)	Reference			
Yes	1.03 (0.51–2.09)	0.927	2.25 (0.63–8.01)	0.212
**Duration of DM diagnosis** (< 5years)	Reference			
≥ 5years	0.79 (0.41–1.54)	0.504	0.78(0.39–1.53)	0.458
**High SBP**(No)	Reference			
Yes	0.58(0.26–1.27)	0.170		
**High DBP**(No)	Reference			
Yes	1.30 (0.42–4.05)	0.623	1.19 (0.37–3.81)	0.677
**HbA1c** (well controlled)	*Reference*			
Poorly controlled	2.13 (1.09–4.54)	**0.049**	2.19 (1.02–4.71)	0.045
**Formal education (YES)**	**Reference**			
NO	1.5 (0.49–5.01)	0.438		
**With history DKA (No)**	Reference			
YES	1.24 (0.64–2.41)	0.523	1.29 (0.66–2.56)	0.454

** -adjusted for Age, Sex, education, duration of diabetes, history of DKA, HbA1C, Blood pressure and Pubertal age

## Discussion

Latent tuberculosis is one of the occurrences in patients with diabetes mellitus globally. With the increase of both diabetes and tuberculosis more so in sub–Saharan Africa, it puts our children and youth with diabetes at the risk of developing both latent and active tuberculosis since sub–Saharan Africa is an endemic area for tuberculosis.

This cross-sectional study shows the high prevalence of latent TB at 14.9%. The prevalence is higher than in developed countries such as Spain (9.1%) [30] and it is lower than what has been reported in Egypt (30-32.1%) [31, 32], South Africa [10], and some non-African countries such as Singapore (28.2%) [33] and Indonesia (38.9%) [34]. The difference in prevalence can be explained by the profound disparity in study setting. To expand further on this point, studies done in high income countries are expected to have lower prevalence compared to low-income countries where TB is endemic. Furthermore, the difference can be credited to the different tests used for TB. Studies that used the Tuberculin Skin Test (TST) might have different prevalence from studies that used QuantiFERON, as QuantiFERON is a more sensitive tool for DM patients [13].

Moreover, studies have shown that poor glycaemic control is among the risk factors for developing Tuberculosis [4, 10]. Unfortunately, studies done in Tanzania and in other parts of Africa (endemic regions for tuberculosis), have high rate of poor glycaemic control posing a great risk of latent tuberculosis and later active tuberculosis [22, 35]. With the current findings, our patients are statistically more susceptible to developing active tuberculosis [10, 24, 36].

After dividing the prevalence of latent tuberculosis by age groups, the pubertal age group [age in median(IQR)][14 (12– 15)] had slightly higher prevalence of latent TB 10 (19.6%) than pre-pubertal age group [Age in Median (IQR)] 8 [6–9] 3 (8.1%) and post-pubertal 29 (15%) age group [Median (IQR)]. The findings in the pubertal age group (median(IQR)][14 (12– 15)]) were similar to the findings shown by Muro et al. and Kidola et al. that had the prevalence of latent tuberculosis at 18% and 19.2% respectively [37, 38]. However, these findings were contrary to other studies where age distribution was similar to all and the age categories (children and youth) [4, 10]. Probably because of the different methods that have been used. We also observed that high proportion (76.2%) of participants with latent Tuberculosis are those who had poor glycaemic control HbA1C > 10%; a finding that is consistent with those from previous studies which showed that poor glycaemic control is a high risk for development of both latent and active tuberculosis [10, 31, 32]. The reason for this is that transient /chronic hyperglycemia modifies the immune system function in patients with DM [32]. Since tuberculosis is an immune reactive disease, it is likely that patients with poor glycaemic control are at high risk of tuberculosis in any form. Type 1 diabetes patients have impaired cellular immunity; hence prone to infections. This underlines the importance of screening for tuberculosis in children and youth with diabetes, so that appropriate therapeutic or prophylactic intervention can be taken early to avoid complications of diabetes. Also, participants with latent tuberculosis in our study were generally older and had a higher weight, this could be attributed to their increased likelihood of being exposed to tuberculosis due to having lived longer.

A patient suffering from latent TB may not be diagnosed or have a delayed diagnosis because they will not have symptoms and those with active tuberculosis with symptoms might not respond well to normal screening methods which are tuberculin skin test, that may appear negative. However QuantiFERON is a better screening tool to help those with latent TB [29]. The WHO recommends screening and treatment of latent tuberculosis in all children under the age of 5 that have been exposed to tuberculosis through a known positive contact [39, 40]. Also, Tanzania is among the 30 high TB burden countries, therefore with this high rate of latent tuberculosis in children and youth with diabetes, screening and prophylaxis in this group might be a viable option; similar to what is done in children under five years and other groups in Tanzania [41, 42].

### Limitation

The study population was only children and youth that are attending pediatric diabetes clinic in Dar es salaam Tanzania, an area with high prevalence of tuberculosis. This treatment, however, is short lasting.

Furthermore, a cross-sectional design was used which cannot demonstrate temporality,

We also did not consider the hemoglobin levels of our participants in relation to HbA1C, hence likelihood of poor glycemic control appearing as good glycaemic control because of their anemia status.

## Conclusion

There is a high prevalence of latent tuberculosis in children and youth with diabetes in Tanzania with a positive association between latent tuberculosis and increase in HbA1c. Therefore, these findings recommend the prioritizing of further screening and co-management, including improving glycaemic control in children and youth living with diabetes.

## Data Availability

The datasets used and/or analyzed during the current study are not available publicly but available from the corresponding author on reasonable request.
